# Antioxidant-Effective Quercetin Through Modulation of Brain Interleukin-13 Mitigates Autistic-Like Behaviors in the Propionic Acid-Induced Autism Model in Rats

**DOI:** 10.1007/s11481-025-10190-w

**Published:** 2025-04-12

**Authors:** Kubilay Doğan Kılıç, Gökçen Garipoğlu, Burak Çakar, Yiğit Uyanıkgil, Oytun Erbaş

**Affiliations:** 1 Faculty of Medicine, Department of Histology and Embryology, Ege University, İzmir, Türkiye; 2 Institute for Tissue Engineering and Regenerative Medicine, Helmholtz Zentrum München, Munich, Germany; 3 Museum Für Naturkunde, Leibniz Institute for Evolution and Biodiversity Science, Berlin, Germany; 4 Faculty of Health Sciences, Department of Nutrition and Dietetic, Bahçeşehir University, Istanbul, Türkiye; 5 Faculty of Medicine, Department of Histology and Embryology, İstinye University, İstanbul, Türkiye; 6 Cord Blood Cell – Tissue Research and Application Center, Ege University, İzmir, Türkiye; 7 Faculty of Medicine, Biruni Research Center (BAMER), Biruni University, Istanbul, Türkiye

**Keywords:** Autism, Brain, Neuroinflammation, Oxidative Stress, Quercetin

## Abstract

Overproduction of reactive oxygen species occurs when inflammation induces oxidative stress in macrophages and microglia, leading to a self-sustaining cycle of cellular damage and neuroinflammation. Oxidative stress and neuroinflammation are well-established contributors to the pathophysiology of autism spectrum disorders, which are associated with impaired neuronal function, neuronal loss, and behavioral deficits. Damaged cells, through microglial activation, release additional inflammatory mediators under conditions of oxidative stress, exacerbating neuronal damage. Quercetin, a powerful dietary antioxidant, has been shown to scavenge free radicals, reduce oxidative stress, and inhibit inflammatory pathways. Given these properties, we hypothesize that quercetin may improve learning and social skills in individuals with autism spectrum disorders by alleviating oxidative stress and reducing brain levels of inflammatory cytokines. In this study, an autism model was established in 30 rats by intraperitoneal injection of 250 mg/kg/day propionic acid (PPA) for five days. The study groups were as follows: Group 1: Normal ontrol (n = 10); Group 2: PPA + saline (PPAS, n = 10); Group 3: PPA + Quercetin (PPAQ, n = 10). All treatments were administered for 15 days. At the end of the treatment, histological and biochemical analyses of brain tissue and behavioral tests related to autistic-like behaviors were performed. Malondialdehyde, tumor necrosis factor-alpha, and interleukin-13 levels in brain homogenates were significantly higher in the PPAS group compared to the control group, indicating elevated oxidative stress and inflammation following PPA exposure. The PPAQ group significantly reduced oxidative stress parameters and inflammatory biomarkers, demonstrating its antioxidant and anti-inflammatory effects. This biochemical improvement was accompanied by preserving Purkinje cells and neuronal populations, significantly reduced in the PPAS group. Moreover, quercetin-treated rats exhibited improved social behavior and learning, which were severely impaired in the PPAS group. These findings, when interpreted together, suggest that quercetin exerts its neuroprotective effects by targeting oxidative stress and neuroinflammation, thereby preventing neuronal cell loss and alleviating behavioral deficits associated with autism spectrum disorders.

**Graphical Abstract:**

**Figure:**
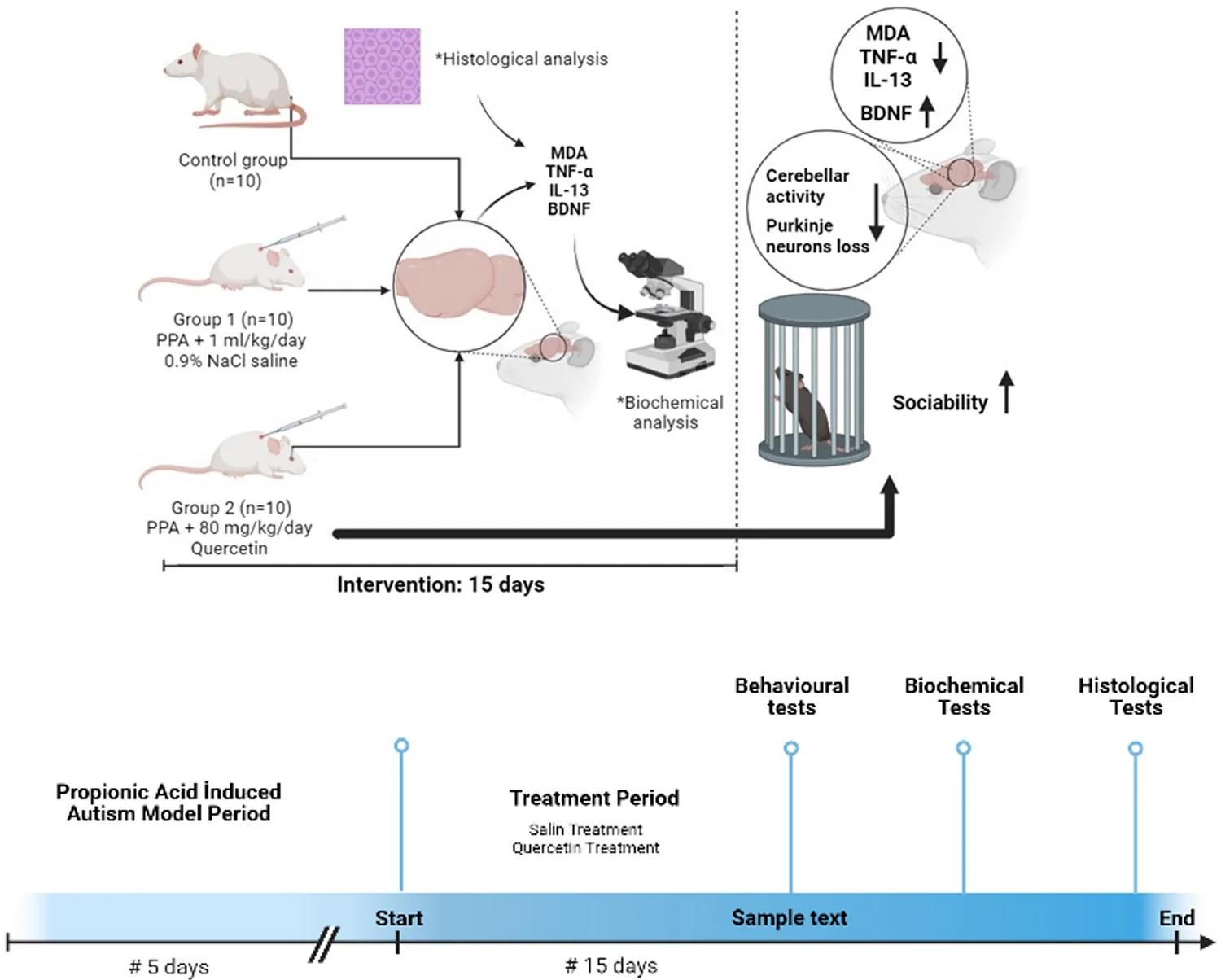

## Introduction

Autism spectrum disorder (ASD) is a neurodevelopmental condition characterized by early-onset social communication difficulties and repetitive sensory-motor behaviors with a wide range of causes, including important genetic factors (Lord et al. 2018; Yang et al. 2023). Recent studies have identified specific genetic mutations, such as in the Shank3 (Uchino and Waga 2013; Zhang et al. 2024), MECP2 (Wen et al. 2017; Karimi et al. 2024), CHD8 (Weissberg and Elliott 2021; Megagiannis et al. 2024). PTEN (Rademacher and Eickholt 2019; Chang et al. 2008) and RELN (Lammert and Howell 2016; Reive et al. 2024) genes, which not only disrupt synaptic function and neurodevelopment but also increase susceptibility to oxidative stress and/or neuroinflammation. These genetic vulnerabilities can impair antioxidant defense systems, leading to chronic oxidative damage and triggering neuroinflammatory responses, which are crucial contributors to ASD pathology.

The pathophysiology of ASD is poorly understood, in part due to its complex and multifaceted etiology involving both environmental and genetic risk factors with differential effects (Özkul et al. 2022; Manivasagam et al. 2020). For instance, mutations in genes such as NRXN1, CACNA1C, and CNTNAP2 have produced diverse phenotypic outcomes depending on the genetic variant and individual susceptibility. While NRXN1 deletions are associated with impaired synaptic signaling and severe social communication deficits (Hu et al. 2019; Bose et al. 2025). CACNA1C variants are linked to altered calcium channel function, affecting both cognitive abilities and motor coordination (Moon et al. 2018; Michels et al. 2018). Similarly, mutations in CNTNAP2 have been correlated with language impairments and repetitive behaviors (Jang et al. 2023; Eve et al. 2022), underscoring the heterogeneity of genetic contributions to ASD. These findings highlight the variability in how genetic predispositions influence ASD development and severity, particularly when combined with environmental factors. Importantly, these genes as mentioned above have also been implicated in pathways related to oxidative stress and/or neuroinflammation, either directly through disrupted cellular signaling or indirectly by impairing antioxidant defense mechanisms, further contributing to ASD pathology.

Oxidative stress and neuroinflammation as well as neurometabolic alterations in specific brain regions are crucial in ASD pathology (Veselinović et al. 2021; Mattaa et al. 2019). Children with ASD typically exhibit high levels of inflammatory biomarkers such as interleukins IL-1β, IL-6, IL-12, tumor necrosis factor-alpha (TNF-α), and interferon alpha omega (IFN-αω), contributing to chronic neuroinflammation and subsequent behavioral abnormalities (Bokobza et al. 2019; Chen, et al. 2021).). Furthermore, increased oxidative stress, particularly in genetically susceptible individuals, has been implicated in ASD pathogenesis, with studies highlighting deficiencies in antioxidant enzymes (Mattos et al. 2020a, b). Genetically susceptible individuals often harbor polymorphisms or mutations in key antioxidant defense genes, such as superoxide dismutase, catalase, and glutathione peroxidase, which are essential for neutralizing ROS and preventing oxidative damage (Carmo et al., 2022). These genetic vulnerabilities lead to impaired enzymatic pathways, resulting in excessive ROS accumulation, lipid peroxidation -as indicated by elevated malondialdehyde (MDA) levels-, and mitochondrial dysfunction. This oxidative stress triggers neuroinflammatory responses, as evidenced by increased levels of pro-inflammatory cytokines such as TNF-α and IL-13 (Kempuraj et al. 2016). Such chronic inflammation further worsens neuronal damage and reduces neurotrophic support, reflected by decreased brain-derived neurotrophic factor (BDNF) levels, contributing to the neuronal loss and behavioral abnormalities observed in ASD pathology (Azman and Zakaria 2022). Although there is no effective drug for ASD, various nutritional factors are also being investigated to support treatment. Studies conducted in populations around the world indicate that there may be deficiencies in antioxidant and methylation metabolites (Liu et al. 2022).

Quercetin, a flavonoid abundant in fruits and vegetables, is widely recognized for its antioxidant and anti-inflammatory effects through its ability to scavenge free radicals and protect against oxidative stress (Karhu et al. 2019; D'Andrea 2015; De Mattos et al. 2020a, b). Notably, quercetin’s capacity to cross the blood–brain barrier makes it a promising candidate for treating neuroinflammatory conditions, including ASD (Tavakoli et al. 2023).

Beyond its role in ASD, quercetin has demonstrated neuroprotective activity by reducing oxidative stress and neuroinflammation in hypoxic-ischemic brain injuries, as seen in studies on cerebral palsy. Its neuroprotective effects are partly mediated through the inhibition of the TLR4/MyD88/NF-κB signaling pathway, which reduces inflammation and improves cognitive and motor outcomes (Le et al. 2020). Moreover, polyphenols, including quercetin, have been shown to modulate oxidative and inflammatory responses in perinatal brain injuries caused by oxygen deprivation, contributing to enhanced neuroplasticity and functional recovery (Pontes et al. 2023). These findings underscore the therapeutic potential of quercetin and related polyphenols in addressing neurodevelopmental disorders characterized by oxidative stress and inflammation.

Further to its direct antioxidant role, quercetin modulates key inflammatory pathways, such as JAK2/STAT3 signaling, which is activated by pro-inflammatory cytokines like IL-6 (Parker-Athill et al. 2009; Bhat and Bhat 2021). By inhibiting this pathway, quercetin reduces neuroinflammation, thereby improving behavioral outcomes in animal models of neurodevelopmental disorders (Kimata et al. 2000; Kilpinen et al. 2008).

Maternal immune activation during pregnancy due to infections or other immune challenges has been implicated in the development of ASD (Zhou et al. 2016). High levels of IL-6 during pregnancy can cause altered fetal brain development, increasing the risk of ASD in offspring (Parker-Athill et al. 2009). In this study, we investigated the effect of quercetin on inflammatory markers and behavioral changes in a rat model of PPA-induced autism. Previous research suggests that quercetin can reduce oxidative damage and improve behavioral parameters in animal models of autism, supporting its potential therapeutic role (De Mattos et al. 2020a, b). The PPA-induced autism model has been widely used to mimic the behavioral and neuroinflammatory characteristics observed in ASD. For instance, studies have shown that PPA administration increases oxidative stress and induces ASD-like behaviors in rodents while elevating pro-inflammatory cytokine levels, such as IL-6, and impairing locomotor activity (MacFabe et al. 2007; Mirza and Sharma 2018). More recent evidence indicates that interventions targeting oxidative stress and inflammation in this model can alleviate neurobehavioral abnormalities, further validating its relevance for ASD research (Hosny et al. 2023; MacFabe et al. 2011). These findings align with our study and highlight the potential of quercetin to modulate neuroinflammatory pathways and improve outcomes in ASD-like conditions.

This research aims to advance the understanding of the neuroprotective effects of quercetin, particularly its ability to modulate brain IL-13 and other inflammatory markers, and provide insights into potential therapeutic strategies for ASD. The findings of this study may pave the way for future clinical trials and the development of dietary interventions to manage ASD symptoms.

## Materials and Methods

### Animals

In this study, 30 male Wistar albino rats aged between 10 and 12 weeks and weighing between 150 and 200 g were used. The experimental protocol was approved by the Animal Ethics Committee of Demiroğlu Bilim University (1223035306). Rats were obtained from a local breeding colony and housed in pairs in steel cages under controlled environmental conditions. The light/dark cycle was maintained at 12/12 h and the temperature was continuously maintained at 22 ± 2 °C.

Rats were provided *ad libitum* food access throughout the experimental period. Throughout the study, the health and welfare of the animals were monitored daily and any signs of distress or disease were immediately addressed according to the guidelines set by the animal ethics committee.

### Experimental Procedures

The experimental design involved the autism model using a total of three litters of Sprague–Dawley rats. Each litter consisted of 10 pups, resulting in 30 rats for the study. Pregnant female Sprague–Dawley rats were obtained from a single breeding colony and housed under controlled environmental conditions (temperature: 22 ± 2 °C; humidity: 50–60%) to ensure consistent breeding. Following delivery, litters were examined for health and genetic viability through physical examination, ensuring no developmental abnormalities or visible deformities.

After weaning, male pups were selected to avoid hormonal influences associated with female offspring. Twenty male rats were used for PPA induction via intraperitoneal injections of 250 mg/kg/day (CheMondis GmbH,79–09-4, Köln, Deutschland)for five consecutive days. An additional ten male rats served as normal controls. The rats were randomly divided into three groups (Jia et al. 2016):

- Group 1 (Control, n = 10): This group received saline solution orally and served as a normal control group without PPA induction.
- Group 2 (PPA + Saline [PPAS], n = 10): This group underwent PPA induction and then received 1 ml/kg/day of 0.9% NaCl saline by oral gavage.
- Group 3 (PPA + Quercetin [PPAQ], n = 10): This group was induced with PPA and then treated with 80 mg/kg/day quercetin (abcam, ab120247, Cambridge, UK) by oral gavage.

The quercetin dose was chosen based on available literature showing that effective doses in humans range from 500 to 1000 mg per day for a period of at least two weeks (Mattos et al. 2020a, b; Andres et al. 2018). The dosage was adjusted appropriately for the rat model to ensure consistency with these findings (Cialdella-Kam et al. 2013; Heinz, et al. 2010).

**Fig. 1:**
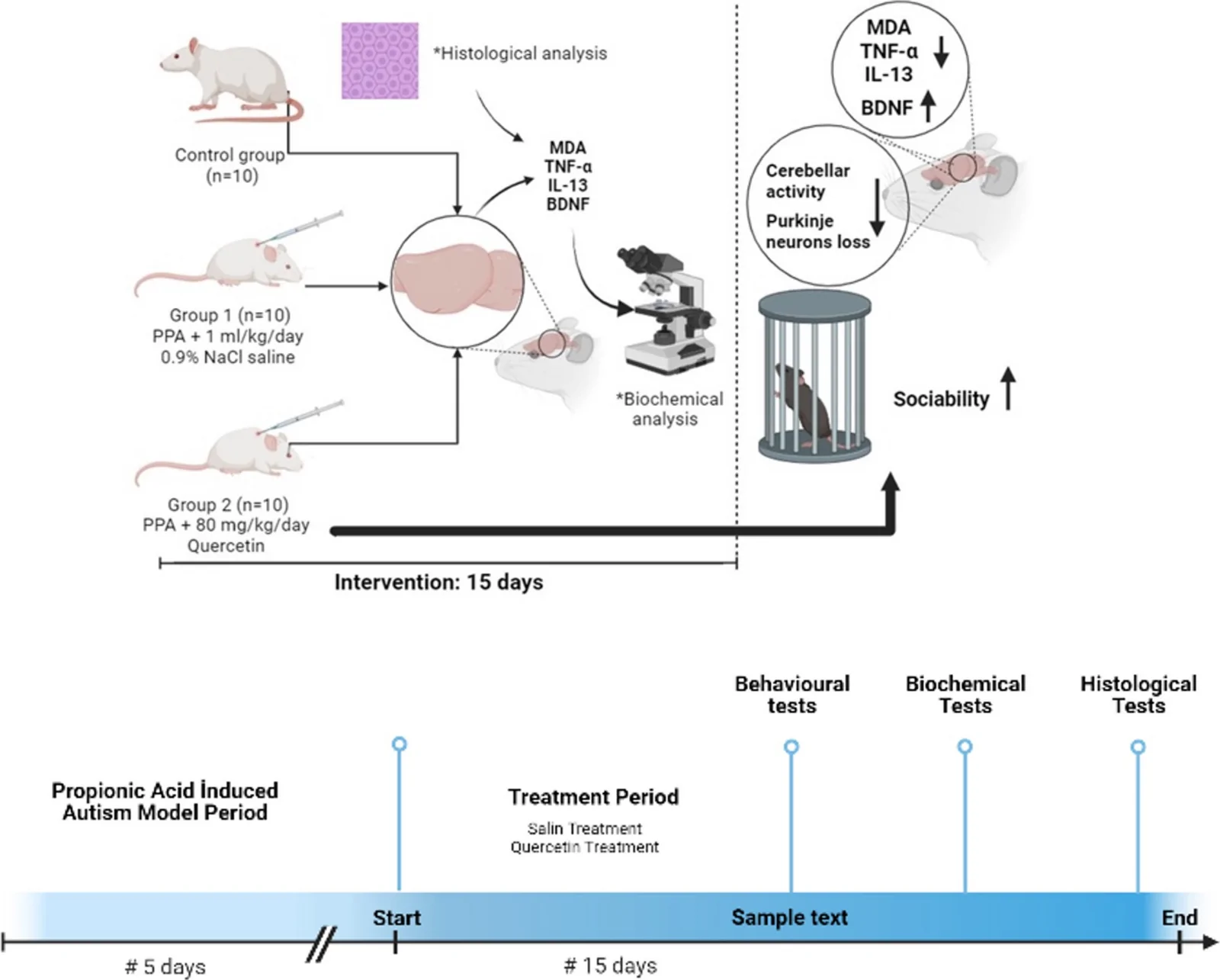
Graphical abstract and experimental timeline

All treatments were administered over fifteen days. To ensure consistency, behavioral assessments were conducted between 10:00 am and 3:00 pm following the treatment period (Fig. 1).

## Behavioral Tests

### Three-Chamber Sociability Test

**Fig. 2:**
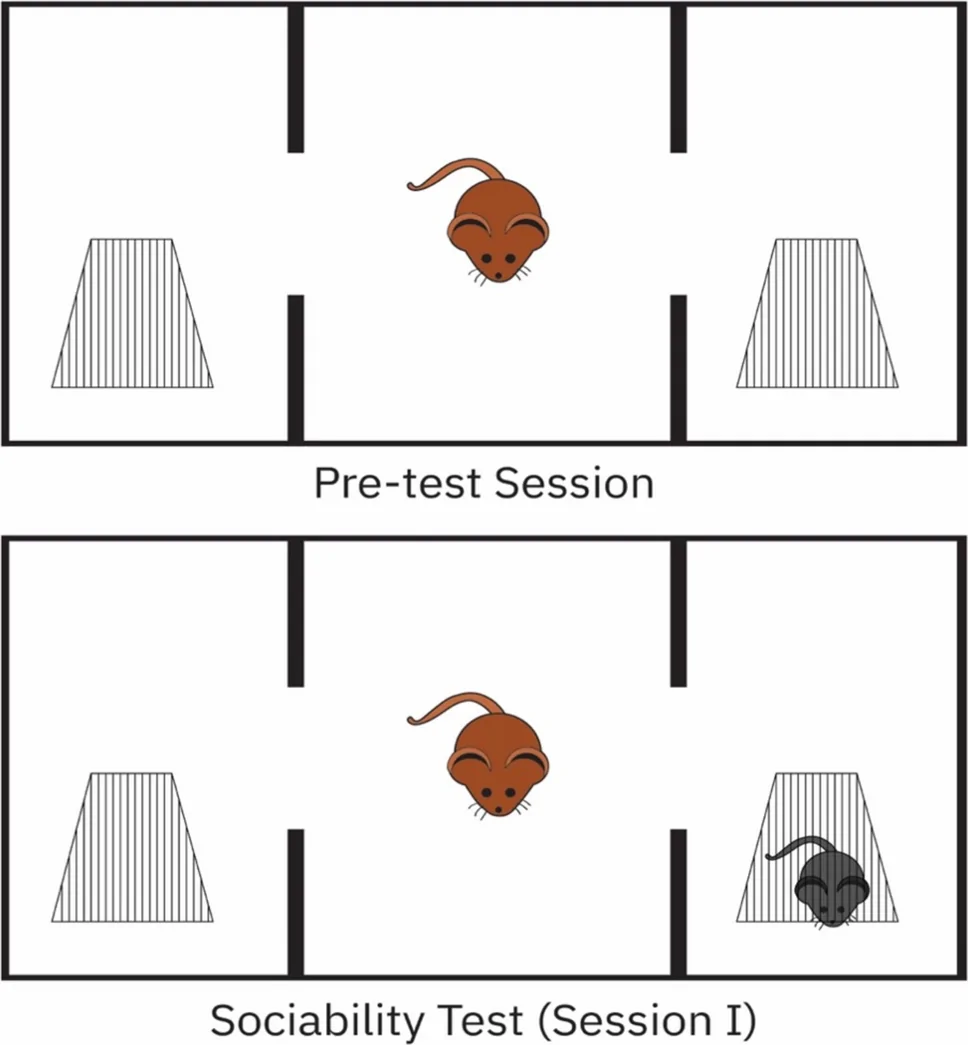
Demonstration of pre-test session and sociability test with Three-Chamber sociability test

A sociability test was performed as previously described with minor modifications (Erbas et al. 2018) Briefly, a Plexiglas cage (40 cm. 90 cm. 40 cm) was divided into three equal regions (40 cm. 30 cm. 40 cm). On the first day, the rats were allowed to habituate in the test cage for 5 min (pre-test session). Twenty-four hours later, to test sociability, a stranger rat (Stranger 1) was placed inside a small plastic cage with mesh-like holes in one side chamber and an empty cage in the third chamber. Then, the test rat was placed in the center chamber and the time spent in each region by the test rat was recorded for 10 min (Session I). The test rat was considered to be in the chamber when its head and two front paws entered the chamber. The floor of the field was then cleaned between each test with 70% alcohol and dried with a paper towel to remove any traces of olfactory stimuli from the previous rat. Time spent with Strangers is calculated as a percentage (Fig. 2).

### Open-Field

The open-field (OF) paradigm is one of the popular behavioral tests to assess locomotion and exploration. Altered OF behavior is relatively simple to observe; however, concluding the reasons for the observed changes is a complex task. Generally, two factors determine the behavior in this paradigm: The first is a positive exploratory drive originating from the nature of rodents to explore new environments (for food and shelter), and the second is the animal nature of avoiding open and brightly lit spaces (exposure to predators). The open-field test is considered useful in determining motor stereotyped behavior, repeated auto grooming, and restriction of research activity in the autism model (Erbas et al. 2018). The open field test was conducted in an open-aired box with dimensions of 50 cm. 50 cm. 40 cm. At the beginning of the test, rats were gently placed in the center of the box and allowed to explore the arena freely for 5 min. Then, each rat was observed for 5 min to evaluate its spontaneous activity level. The total number of ambulation (i.e., the number of floor divisions crossed with four paws) was recorded. The floor of the field was then cleaned between each animal with a 70% alcohol-water solution and dried with a paper towel to avoid olfactory cues.

### Passive Avoidance Learning

Learning and memory performance of offspring were evaluated by passive avoidance learning (PAL) test as FCA described previously (Erbas et al. 2014). PAL is comprised of fear-motivated avoidance tasks in which the rat learns to refrain from stepping through a door that seems safer but leads into a dark compartment with an electrified grid system that delivers a shock. The PAL box was 20 cm × 20 cm × 20 cm and had both dark and lighted chambers. Normally when a rat was placed into the lighted compartment, they preferred to enter the dark chamber. After a 10-s habituation period in the lighted compartment, the guillotine door separating the light and dark chambers was opened. When a rat passed into the dark chamber, the door separating the light and dark compartments was closed. Then a 1.5 mA electric shock was delivered over 3 s, and the rat was subsequently removed from the dark chamber and returned to its cage. Twenty-four hours later, the rats were placed into the PAL box again. The duration of time or latency period for the rat to travel from the light to the dark chamber was recorded, but a shock was not delivered. The latency period was recorded up to a maximum of 300 s. The time that the rat took to refrain from crossing into the dark chamber served as an index of the rat's memory.

### Histochemistry

To assess neuronal density and evaluate morphological changes in brain tissues, hematoxylin and eosin (H&E) staining was performed. Brain tissues were fixed in 10% formalin (Sigma-Aldrich, HT501128, Massachusetts, USA) for 48 h, dehydrated through a graded ethanol series (Merck, 64–17-5, Darmstadt, Germany), cleared in xylene (VWR Chemicals, 1330–20-7, Mississauga, Canada), and embedded in paraffin wax (Leica Biosystems, 3801320, Illinois, USA). Coronal sections of 5 µm thickness were obtained using a rotary microtome and mounted onto glass slides. The sections were deparaffinized in xylene and rehydrated in decreasing concentrations of ethanol to distilled water.

For H&E staining, the sections were first stained with hematoxylin (Merck, 517–28-2, Darmstadt, Germany) to visualize cell nuclei, followed by counterstaining with eosin (Sigma-Aldrich, HT110216, Massachusetts, USA) to highlight cytoplasmic and extracellular matrix components. Stained sections were dehydrated, cleared, and mounted using a permanent mounting medium (Thermo Fisher Scientific, SP15-500, Waltham, USA).

Neuronal counts were assessed in hippocampal CA1 and CA3 regions as well as in the cerebellar Purkinje cell layer. Histological evaluation was performed using a light microscope (Olympus, Olympus BX51, Tokyo, Japan) at 20 × and 40 × magnifications. Images were digitally captured using a high-resolution camera (Olympus, Olympus C-5050, Tokyo, Japan). Quantitative analysis of neuronal density was carried out using ImageJ software (version 4.0.21), following standard protocols for manual cell counting within defined regions of interest. This histochemical analysis allowed for the precise quantification of neuronal populations and provided critical insights into the protective effects of quercetin against PPA-induced neuronal loss.

### Immunohistochemistry

The process involved the preparation of brain tissues for histological analysis by fixation, sectioning, and staining. Brain tissues were fixed in 10% formalin (Sigma-Aldrich, HT501128, Massachusetts, USA) for 48 h, dehydrated in ethanol (Merck, 64–17-5, Darmstadt, Germany) cleared in xylene(VWr Chemicals, 1330–20-7, Mississauga Canada), and embedded in paraffin wax (Leica Biosystems, 3801320, Illinois, USA). Coronal sections of 5 µm thickness were taken using a rotary microtome and mounted on glass slides. Sections were deparaffinized in xylene and rehydrated in a decreasing series of ethanol to distilled water.

For immunohistochemical staining, glial fibrillary acidic protein (GFAP) was targeted to assess glial activity and neuronal integrity, respectively. Antigen retrieval for GFAP staining was performed by heating the sections in a citrate buffer (abcam, ab93678, Cambridge, UK) (10 mM, pH 6.0) at 90 °C for 30 min. Sections were treated with 3% hydrogen peroxide in methanol for 10 min to quench endogenous peroxidase activity and then blocked with 5% normal goat serum (abcam, ab7481, Cambridge, UK) in phosphate-buffered saline (PBS) (Sigma-Aldrich, P4417, Massachusetts, USA) for 30 min to prevent non-specific binding. Sections were then incubated with rabbit polyclonal anti-GFAP antibody abcam, ab7260, Cambridge, UK) (1:200 dilution) overnight at 4 °C, followed by incubation with biotinylated goat anti-rabbit IgG secondary antibody (abcam, ab6720, Cambridge, UK) (1:400 dilution) for 30 min at room temperature. Sections were treated with Vectastain ABC reagent (Vector Laboratories, PK-4000, California, USA)for 30 min and immunoreactivity was visualized using diaminobenzidine (DAB) (abcam, ab64238, Cambridge, UK) as chromogen and hematoxylin (Merck, 517–28-2, Darmstadt, Germany) as counterstain. Sections were prepared for IHC examinations by the recommendations of the manufacturers of the antibodies in the study.

Immunostained sections were examined under a light microscope (Olympus, Olympus BX51, Tokyo, Japan) and images were captured digitally (Olympus, Olympus C-5050, Tokyo, Japan). Quantitative analysis of GFAP immunoreactivity was performed using ImageJ software (version 4.0.21), which included measuring the area fraction of GFAP-positive staining. This comprehensive immunohistochemical analysis provided insights into the neuroprotective effects of quercetin on astrocyte activity and neuronal survival in a PPA-induced autism model.

### Biochemical Analysis

At the end of the study, all animals were humanely sacrificed by cervical dislocation under sedation using ketamine (100 mg/kg, Ketasol, Richterpharma AG, Austria) and xylazine (50 mg/kg, Rompun, Bayer, Germany). Blood samples were collected via cardiac puncture for biochemical analysis. Brain tissues were homogenized in ice-cold PBS containing protease inhibitors (Sigma-Aldrich, P4417, Massachusetts, USA) to prevent protein degradation.

Homogenates were centrifuged at 10,000 g for 15 min at 4 °C to remove cell debris and the supernatant was collected for further analysis (Sigma Laborzentrifugen, SIGMA-3K30, Osterode am Harz.

Germany). MDA (abcam, E-EL-0060, Cambridge, UK), TNF-α (abcam, E-EL-R2856, Cambridge, UK), interleukin-13 (IL-13) (abcam, E-EL-R0563, Cambridge, UK) and BDNF (abcam, E-EL-R1235, Cambridge, UK) levels were measured using enzyme-linked immunosorbent assay (ELISA) kits according to the manufacturer's instructions. MDA levels, an indicator of lipid peroxidation and oxidative stress, were measured using a commercial Thiobarbituric Acid Reactive Substances assay kit (abcam, E-BC-K298-M, Cambridge, UK) and absorbance was read at 532 nm. MDA concentrations were expressed as nmol/mg protein.

To evaluate the inflammatory response, TNF-α and IL-13 levels were measured using rat-specific ELISA kits and absorbance was read at 450 nm. The concentrations of these cytokines were expressed as pg/mg protein. BDNF levels, which serve as a marker of neurotrophic support, were also measured using a rat-specific BDNF ELISA kit, absorbance was read at 450 nm and concentrations were expressed as pg/mg protein.

Specimens were prepared for ELISA by the recommendations of the manufacturers of the antibodies in the study.

Protein concentrations in brain homogenates were determined using the Bradford protein assay. Absorbance was read at 595 nm and protein concentrations were calculated using a bovine serum albumin standard curve for calibration expressed in mg/mL. The biochemical analysis aimed to elucidate the effects of quercetin on oxidative stress, inflammatory markers, and neurotrophic factors in the brain tissue of rats in a PPA-induced autism model.

### Statistical Analysis

Data were analyzed using the Statistical Package for Social Sciences (SPSS) version 15.0. Results are presented as mean ± standard deviation (SD). The normality of data distribution was assessed using the Shapiro–Wilk test, while the homogeneity of variance was assessed using the Levene test.

One-way analysis of variance (ANOVA) was used to compare differences between the control, PPAS, and PPAQ groups. When ANOVA showed significant differences, post hoc comparisons were made using the Tukey Honestly Significant Difference (HSD) test to determine which groups were significantly different. The Kruskal–Wallis test was used for nonparametric data, followed by pairwise comparisons with Bonferroni correction. Statistical significance was set at p < 0.05 for all analyses.

## Results

### Behavioral Tests

#### Three-Chamber Sociability Test

The three-chamber sociability test assessed the social interaction abilities of rats. The percentage of time spent interacting with the unfamiliar rat was significantly reduced in the PPAS (34.1 ± 1.13%) compared to the control group (64.3 ± 2.9%). One-way ANOVA showed a significant difference among groups (F(2,27) = 45.2, p < 0.001), and post-hoc comparisons revealed a significant reduction in the PPA + saline group compared to controls (**p = 0.0008; p < 0.001). However, the PPAQ group (60.8 ± 1.7%) spent significantly more time interacting with the unfamiliar rat compared to the PPAS group (p = 0.0012; #p < 0.05), indicating that quercetin treatment improved sociability.

These results demonstrate that quercetin effectively attenuates social deficits induced by PPA exposure, restoring sociability levels close to those observed in the control group.

#### Open-Field Test

The open-field test measured exploratory behavior and anxiety levels. The number of crossings in the open field was significantly reduced in the PPA + saline group (6.2 ± 2.5) compared to the control group (19.8 ± 3.3). One-way ANOVA revealed a significant difference among the groups (F(2,27) = 18.6, p < 0.001), with post-hoc comparisons showing a significant reduction in the PPA + saline group compared to controls (**p = 0.0005; p < 0.001). However, the PPA + quercetin group (14.2 ± 3.8) showed a significant increase in the number of crossings compared to the PPA + saline group (p = 0.012; #p < 0.05), indicating that quercetin treatment improved exploratory activity and reduced anxiety.

These results suggest that quercetin may have anxiolytic effects and promote normal exploratory behavior in the PPA-induced autism model.

#### Passive Avoidance Learning

The passive avoidance test assessed learning and memory abilities. The latency to avoid the dark compartment was significantly shorter in the PPAS group (89.3 ± 13.8 s) compared to the control group (244.9 ± 10.6 s), as determined by one-way ANOVA (F(2,27) = 26.7, p < 0.001). Post-hoc analysis showed a significant difference between the PPAS group and the control group (**p = 0.0003; p < 0.001), indicating impaired learning and memory due to PPA exposure.

In contrast, the PPAQ group (189.6 ± 27.5 s) exhibited a significantly longer latency to avoid the dark compartment compared to the PPAS group (p = 0.024; #p < 0.05), suggesting that quercetin treatment improved learning and memory abilities.

This improvement in cognitive function suggests that quercetin may compensate for cognitive deficits induced by PPA. Comprehensive biochemical, histological, and behavioral analyses demonstrate the beneficial effects of quercetin in reducing oxidative stress, reducing inflammation, preserving neuronal integrity, and improving behavioral outcomes in a PPA-induced autism model. These findings suggest that quercetin has potential as a therapeutic agent for managing autism spectrum disorder and its associated symptoms (Table 1).

**Table 1:** Behavioral test results

	Normal Control	PPA + saline	PPA + Quercetin
Sociability test: The spend of time with stranger rat percent (%)	64.3 ± 2.9	34.1 ± 1.13*	60.8 ± 1.7##
Open Field Test: Number of ambulation	19.80 ± 3.3	6.2 ± 2.5**	14.2 ± 3.8##
Passive avoidance learning (PAL) Latency (Sec.)	244.9 ± 10.6	89.3 ± 13.8*	189.6 ± 27.5#
Sociability test: The spend of time with stranger rat percent (%)	64.3 ± 2.9	34.1 ± 1.13**	60.8 ± 1.7##
Open Field Test: Number of ambulation	19.80 ± 3.3	6.2 ± 2.5**	14.2 ± 3.8#
Passive avoidance learning (PAL) Latency (Sec.)	244.9 ± 10.6	89.3 ± 13.8**	189.6 ± 27.5#

Results were presented as mean ± SEM. Statistical analyses were performed by one-way ANOVA. **p < 0.001 different from normal groups; #p < 0.05, ##p < 0.001 different from PPA and saline group.

#### Immunohistochemical and Histochemical Analysis

**Fig. 3:**
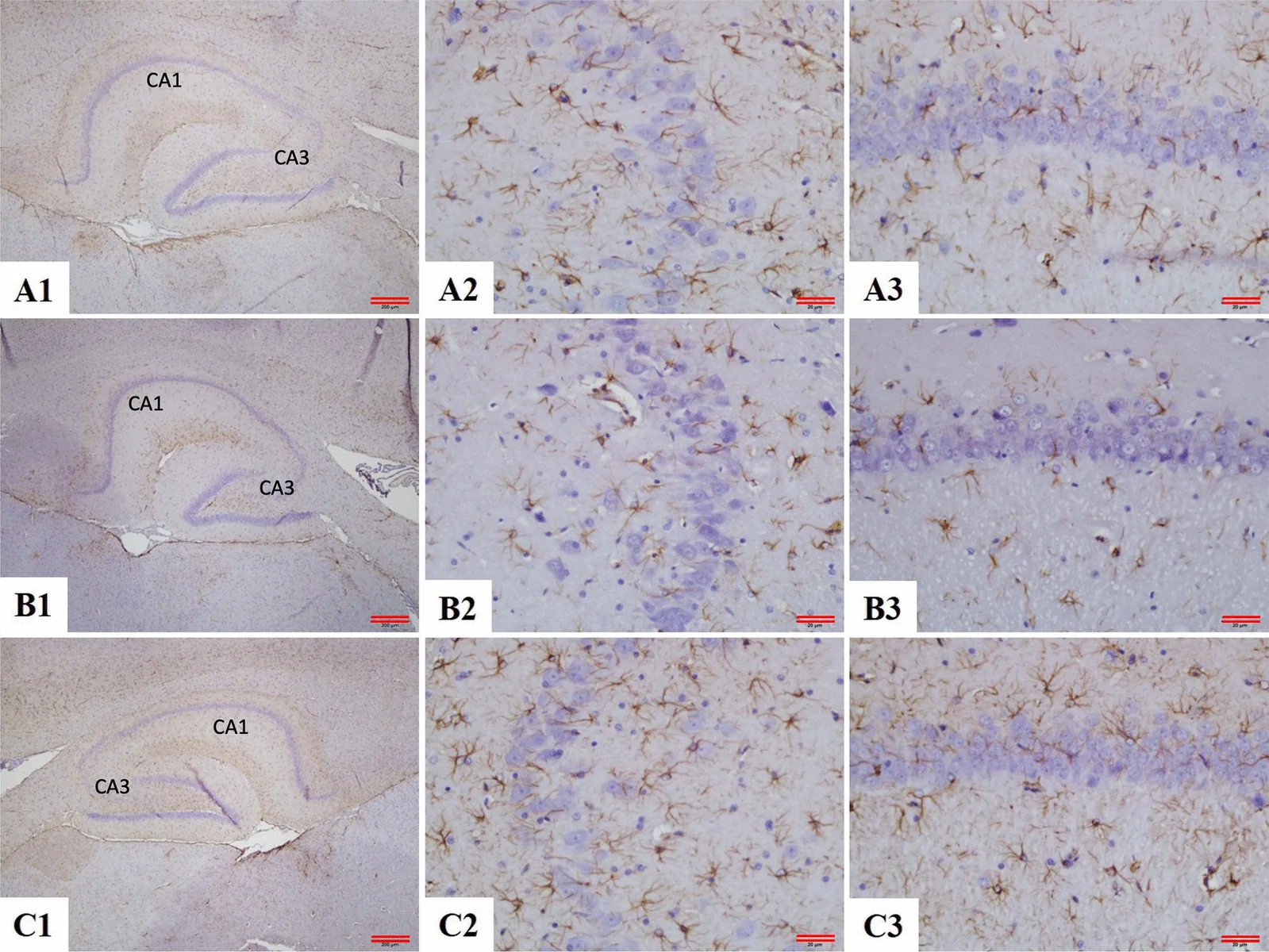
Immunohistochemical Staining of GFAP in Hippocampal CA1 and CA3 Regions. Control group male rats show minimal GFAP-positive staining, indicating normal astrocyte activity and low levels of astrocytosis. (A1) At 10 × magnification, the control group shows minimal GFAP-positive staining, while (A2) at 40 × magnification in CA1 and (A3) at 40 × magnification in CA3, the sparse presence of GFAP is specific to healthy brain tissue, confirming a stable astrocyte environment. The PPA + Saline (PPAS) group male rats exhibit increased GFAP staining (B1), highlighting significant astrocyte activation and gliosis in response to PPA. (B2) At 40 × magnification, the PPAS group shows increased GFAP staining in CA1, and (B3) at 40 × magnification, the extent of astrocytic hypertrophy and increased GFAP expression is more evident, indicating severe neuroinflammation in CA3. The PPA + Quercetin (PPAQ) group male rats show a reduction in GFAP staining compared to the PPAS group (C1). At 40 × magnification, quercetin treatment reduces astrocytosis, and at 40 × magnification, the decrease in astrocyte activation is more pronounced in CA1 and CA3 (C2-C3), indicating the neuroprotective effects of quercetin. GFAP immunoreactivity was visualized using DAB as a chromogen, and sections were counterstained with hematoxylin. The scale bar represents 50 µm in 10 × images and 20 µm in 40 × images

**Fig. 4:**
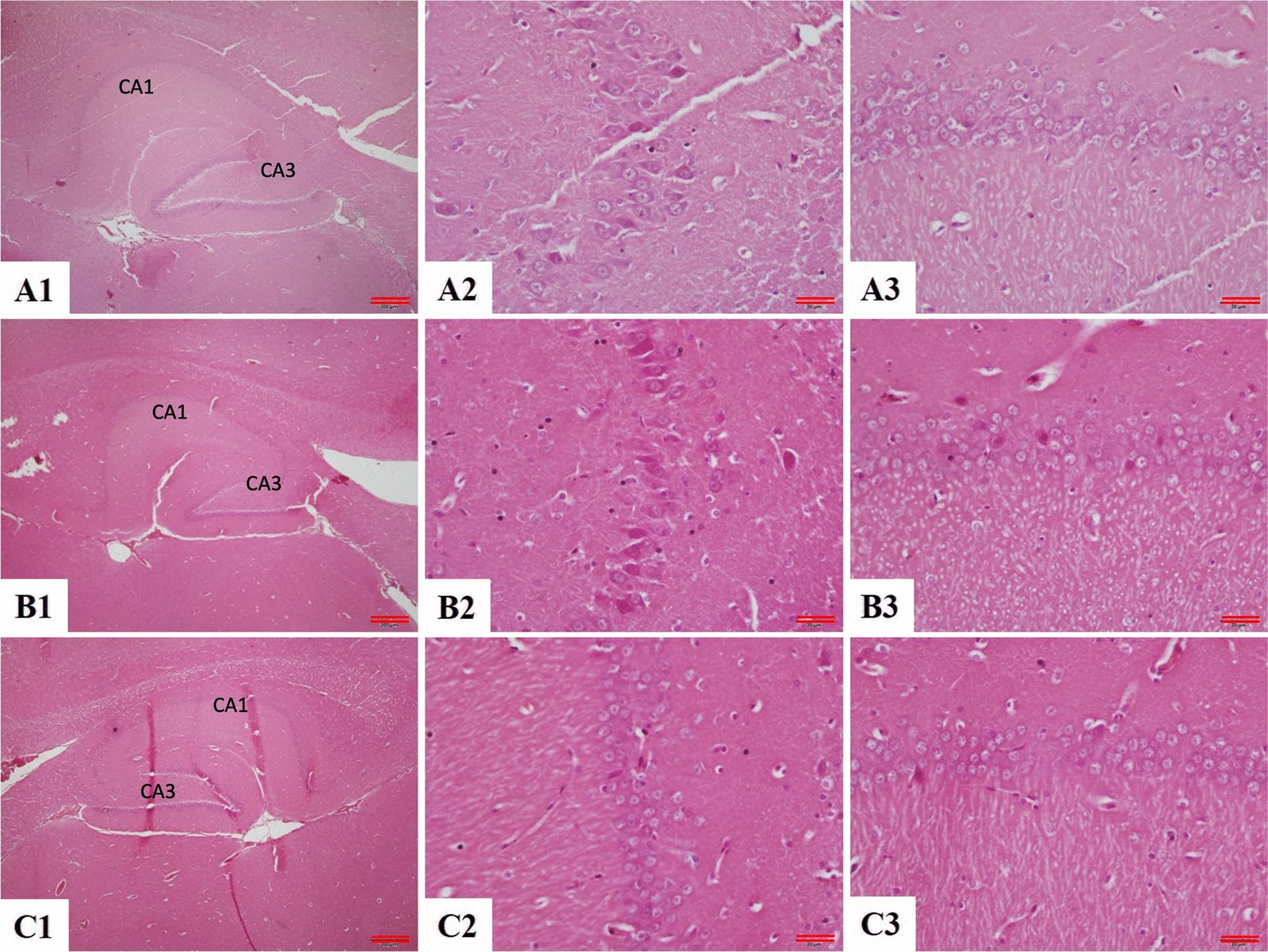
H&E Staining and Neuronal Counts in the Hippocampal CA1 and CA3 Regions. Control group male rats show preserved hippocampal architecture, intact pyramidal cell layers, and a high neuronal density. (A1) At 10× magnification, the control group shows the general architecture of the hippocampus, while (A2) at 40× magnification in CA1 and (A3) at 40× magnification in CA3, neurons exhibit intact nuclei and preserved cellular morphology. The PPA+Saline (PPAS) group male rats exhibit reduced neuronal density and morphological alterations associated with PPA-induced neuronal damage (B1). At 40× magnification, the PPAS group shows reduced neuronal density and disrupted cellular organization in CA1 (B2) and CA3 (B3). The PPA+Quercetin (PPAQ) group male rats show preservation of hippocampal architecture and neuronal density compared with the PPAS group (C1). At 40× magnification, quercetin treatment preserves neuronal morphology and cellular organization in CA1 (C2) and CA3 (C3), indicating its neuroprotective effects against PPA-induced hippocampal damage. Quantitative neuronal counts are presented in Table 2. Scale bars represent 200 µm in panels A1, B1, and C1 and 20 µm in panels A2–A3, B2–B3, and C2–C3

GFAP immunostaining was performed to assess glial activity and astrocyte response in the hippocampus (Fig. 3). In the CA1 region, the PPAS group exhibited a significantly higher GFAP-positive staining area (45.8 ± 1.2) compared to the control group (34.8 ± 1.5; p = 0.006; p < 0.01), indicating increased astrocyte activation and gliosis due to PPA-induced injury. Quercetin treatment significantly reduced GFAP expression in the PPAQ group (33.8 ± 0.9) compared to the PPAS group (p = 0.0005; p < 0.001), suggesting attenuation of gliosis. Similarly, in the CA3 region, GFAP staining was significantly elevated in the PPAS group (42.5 ± 1.7) compared to the control group (30.4 ± 1.5; p = 0.0002; p < 0.001). Quercetin treatment significantly reduced GFAP levels in the PPAQ group (33.1 ± 2.2) compared to the PPAS group (p = 0.021; p < 0.05). In the CA1 region, the PPAS group showed a significant reduction in neuronal count (60.1 ± 2.6) compared to the control group (79.7 ± 1.4; p = 0.004; p < 0.01). Quercetin treatment significantly improved the number neurons in the PPAQ group (73.1 ± 0.8) compared to the PPAS group (p = 0.028; p < 0.05). Similarly, in the CA3 region, neuronal count was significantly lower in the PPAS group (32.5 ± 0.6) compared to the control group (47.5 ± 1.4; p = 0.013; p < 0.05). Quercetin treatment significantly increased neuronal survival in the PPAQ group (40.3 ± 2.0) compared to the PPAS group (p = 0.032; p < 0.05) (Fig. 4).

In the cerebellum, GFAP-positive staining was significantly higher in the PPAS group (26.8 ± 1.5) compared to the control group (15.3 ± 0.8; p = 0.007; p < 0.01). Quercetin treatment significantly reduced GFAP expression in the PPAQ group (20.4 ± 0.9) compared to the PPAS group (p = 0.018; p < 0.05).

**Fig. 5:**
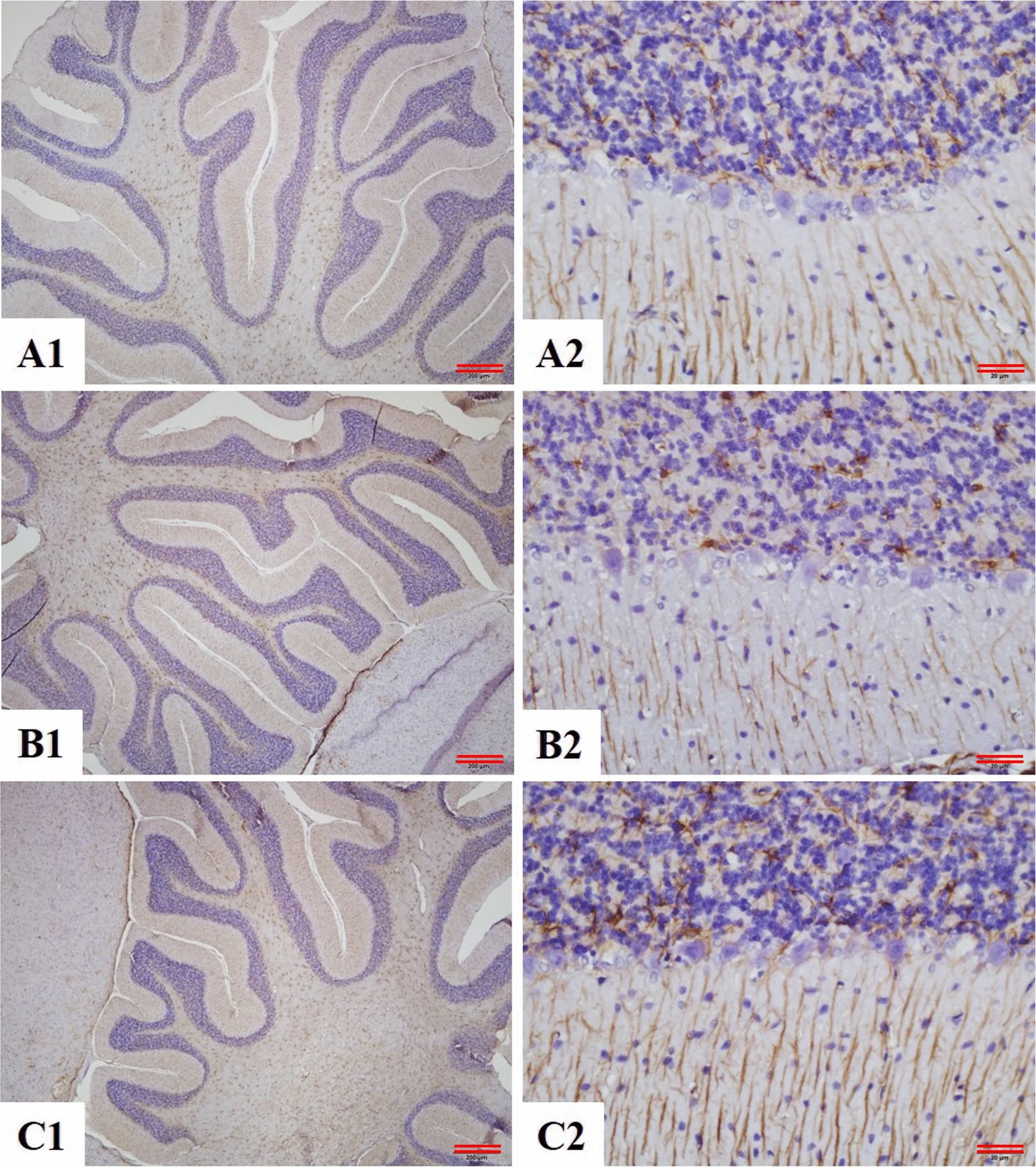
(A1-A2) Control group male rats show minimal GFAP-positive staining, indicative of normal astrocyte activity and low levels of astrocytosis. (A1) At 10 × magnification, GFAP-positive staining is sparse and characteristic of a healthy astrocytic environment, while (A2) at 40 × magnification, the absence of significant GFAP-positive hypertrophy in Purkinje layers confirms stable astrocytic activity. (B1-B2) The PPA + Saline (PPAS) group male rats exhibit significantly elevated GFAP-positive staining, indicative of increased astrocyte activation and gliosis due to PPA exposure. (B1) At 10 × magnification, GFAP staining reveals marked astrocytic activation, and (B2) at 40 × magnification, the extent of gliosis and astrocytic hypertrophy is more evident in Purkinje cell layers. (C1-C2) The PPA + Quercetin (PPAQ) group male rats demonstrate a reduction in GFAP-positive staining compared to the PPAS group. (C1) At 10 × magnification, quercetin treatment reduces astrocytosis, while (C2) at 40 × magnification, astrocyte activation in Purkinje layers is significantly reduced, resembling levels seen in the control group. These findings emphasize quercetin's neuroprotective effects in mitigating astrocytic hypertrophy and inflammation. GFAP immunoreactivity was visualized using DAB as a chromogen, and sections were counterstained with hematoxylin. The scale bar represents 50 µm in 10 × images and 20 µm in 40 × images

**Fig. 6:**
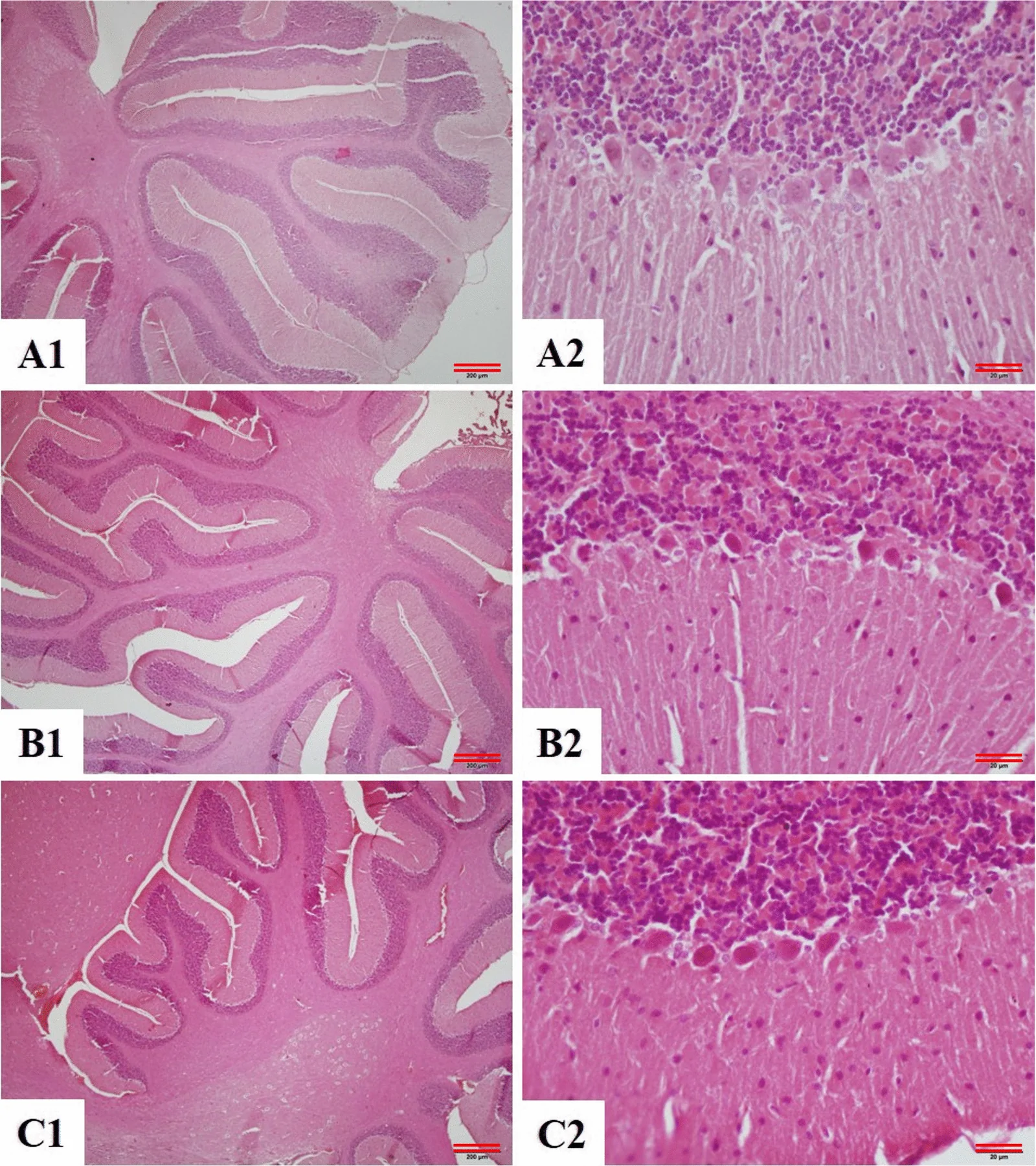
H&E Staining and Neuronal Counts in the Cerebellum. The neuronal density in Purkinje layers of the cerebellum was assessed using H&E staining. Control group male rats exhibited high neuronal density with clearly defined Purkinje cell layers, indicating intact neuronal integrity. In contrast, the PPA + Saline (PPAS) group showed a significant reduction in neuronal density, with disrupted Purkinje morphology and evidence of degeneration, indicative of PPA-induced neuronal damage. H&E staining revealed disorganized cell layers and reduced neuronal populations in the Purkinje cell layers. Quercetin treatment in the PPA + Quercetin (PPAQ) group significantly preserved neuronal density and restored Purkinje cell layer morphology, demonstrating quercetin's neuroprotective potential against PPA-induced neurotoxicity. Quantitative analysis confirmed these observations, underscoring the effectiveness of quercetin in maintaining cerebellar neuronal integrity and reducing neuroinflammation

GFAP immunostaining was used to evaluate neuronal integrity and survival in the cerebellum via astocytes (Fig. 5). The Purkinje cell count was significantly reduced in the PPAS group (12.2 ± 1.2) compared to the control group (25.2 ± 1.7; p = 0.0001; p < 0.001). Quercetin treatment significantly improved the Purkinje cell count in the PPAQ group (20.4 ± 1.6) compared to the PPAS group (p = 0.017; p < 0.05) (Fig. 6).

These findings collectively demonstrate that PPA exposure induces significant neuronal loss, gliosis, and Purkinje cell degeneration in hippocampal and cerebellar regions. Quercetin treatment effectively attenuates these changes, likely through its antioxidant and anti-inflammatory properties, preserving neuronal populations and reducing astrocyte activation (Table 2).

**Table 2:** Merged and normalized cell counting results

	Normal Control	PPA + saline	PPA + Quercetin
Neuronal Count CA1	79.7 ± 1.4	60.1 ± 2.6**	73.1 ± 0.8#
Neuronal Count CA3	47.5 ± 1.4	32.5 ± 0.6*	40.3 ± 2.0#
GFAP immunostaining index (CA1)	34.8 ± 1.5	45.8 ± 1.2	33.8 ± 0.9##
GFAP immunostaining index (CA3)	30.4 ± 1.5	42.5 ± 1.7**	33.1 ± 2.2#
Purkinje Count Cerebellum	25.2 ± 1.7	12.2 ± 1.2**	20.4 ± 1.6#
GFAP immunostaining index (Cerebellum)	15.3 ± 0.8	26.8 ± 1.5*	20.4 ± 0.9#

Results were presented as mean ± SEM. Statistical analyses were performed by one-way ANOVA followed by post-hoc comparisons. Statistical significance was determined as follows: p < 0.01 (*), p < 0.001 (**) compared to the control group; p < 0.05 (#), p < 0.001 (##) compared to the PPA + saline group.

#### Biochemical Analysis

Biochemical analysis revealed significant differences in oxidative stress, inflammatory cytokines, and neurotrophic factors among the experimental groups, highlighting the neuroprotective effects of quercetin (Table 3).

**Table 3:** Biochemical analyses results

	Normal Control	PPA + saline	PPA + Quercetin
Brain MDA level (nmol/g protein)	59.9 ± 3.05	127.3 ± 5.5**	66.2 ± 7.01##
Brain TNF-α level (pg/mg protein)	11.6 ± 0.9	133.8 ± 7.4**	73.4 ± 2.9##
Brain IL-13 level (pg/mg protein)	14.2 ± 4.4	46.1 ± 1.8*	26.8 ± 1.5#
Brain BDNF level (pg/mg protein)	14.1 ± 1.6	7.5 ± 0.8*	12.5 ± 1.1#

MDA levels as an oxidative stress parameter, were significantly elevated in the PPAS group (127.3 ± 5.5 nmol/g protein) compared to the control group (59.9 ± 3.05; p = 0.0001; p < 0.001), indicating that PPA exposure led to increased lipid peroxidation and oxidative damage. Quercetin treatment significantly reduced MDA levels in the PPAQ group (66.2 ± 7.01) compared to the PPAS group (p = 0.0003; p < 0.001), demonstrating quercetin's antioxidant capacity in alleviating oxidative stress by scavenging free radicals.

TNF-α levels, a key pro-inflammatory cytokine, were significantly increased in the PPAS group (133.8 ± 7.4 pg/mg protein) compared to the control group (11.6 ± 0.9; p = 0.0001; p < 0.001). Quercetin treatment significantly reduced TNF- α levels in the PPAQ group (73.4 ± 2.9) compared to the PPAS group (p = 0.0002; p < 0.001), highlighting its potent anti-inflammatory effect, likely mediated through inhibition of pro-inflammatory signaling pathways. IL-13 levels, another cytokine involved in inflammatory responses, were significantly elevated in the PPAS group (46.1 ± 1.8 pg/mg protein) compared to the control group (14.2 ± 4.4; p = 0.007; p < 0.01). Quercetin treatment significantly decreased IL-13 levels in the PPAQ group (26.8 ± 1.5) compared to the PPAS group (p = 0.015; p < 0.05), demonstrating its role in modulating immune responses and reducing inflammation.

BDNF levels, a neurotrophic factor critical for neuronal survival and plasticity, were significantly decreased in the PPAS group (7.5 ± 0.8 pg/mg protein) compared to the control group (14.1 ± 1.6; p = 0.012; p < 0.05). Quercetin treatment significantly restored BDNF levels in the PPAQ group (12.5 ± 1.1) compared to the PPAS group (p = 0.032; p < 0.05), suggesting a neuroprotective role of quercetin in enhancing neuronal survival and synaptic plasticity.

Results were presented as mean ± SEM and statistical analyses were performed using one-way ANOVA followed by post-hoc comparisons. Statistical significance was indicated as follows: p < 0.01 (*), p < 0.001 (**) compared to the control group; p < 0.05 (#), p < 0.001 (##) compared to the PPA + saline group.

## Discussion

The present study investigated the neuroprotective effects of quercetin on oxidative stress, inflammation, neuronal integrity, and behavioral outcomes in a PPA-induced autism model in rats. The findings indicate that quercetin significantly reduced oxidative damage, reduced inflammatory markers, preserved neuronal integrity, and improved social and cognitive behaviors. The high MDA levels in the PPAS group confirmed the role of oxidative stress in the PPA-induced autism model, which is consistent with previous studies reporting increased oxidative stress markers in ASD (Chauhan and Chauhan 2006; Lobzhanidze et al. (2018). Quercetin administration in the PPAQ group significantly reduced MDA levels, highlighting its potent antioxidant properties. Quercetin's ability to scavenge free radicals and inhibit lipid peroxidation is well documented (Boots et al. 2008), suggesting that it effectively combats oxidative stress, a key pathological feature in ASD. Inflammatory cytokines such as TNF-α and IL-13 were significantly elevated in the PPAS group, indicating a strong inflammatory response after PPA administration (Tobiasova et al. 2011; Saghazadeh et al. 2019). These results are consistent with existing literature showing elevated pro-inflammatory cytokines in individuals with ASD (Ashwood et al. 2011). The significant decrease in TNF-α and IL-13 levels in the PPAQ group highlights the anti-inflammatory effects of quercetin, which is known to regulate inflammatory pathways by inhibiting the activation of nuclear factor kappa B (NF-κB) and other pro-inflammatory signaling molecules (Nair et al. 2006). This anti-inflammatory effect likely contributes to the neuroprotective effects observed in this study (Al-Beltagi 2021). The connection between inflammation and autism-like behaviors is well-established, particularly in animal research (Doğan et al. 2023).

The preservation of neuronal integrity, as indicated by the higher number of neuron numbers in the PPAQ group, further supports the neuroprotective role of quercetin. The reduction in neuronal loss in the hippocampal CA1 and CA3 regions in the PPAQ group suggests that quercetin may protect against PPA-induced neurodegeneration. This is consistent with previous studies showing the neuroprotective effects of quercetin in various models of neurodegeneration (Spencer 2010). The improvement in neuronal survival may be attributed to the antioxidant and anti-inflammatory properties of quercetin, which helps attenuate the deleterious effects of oxidative stress and inflammation on neurons. Behavioral tests revealed significant improvements in social interaction, exploratory behavior, and cognitive function in the PPAQ group compared to the PPAS group. In the three-chamber sociability test, quercetin-treated rats spent more time with an unfamiliar rat, indicating improved sociability, an important finding considering that social deficits are a core feature of ASD. The open field test showed increased exploratory behavior and decreased anxiety in the PPAQ group, suggesting that quercetin may have anxiolytic effects. The PAL test showed improved learning and memory in quercetin-treated rats, further supporting its potential cognitive benefits. These behavioral improvements are likely related to the neuroprotective and anti-inflammatory effects of quercetin, which help preserve neuronal function and plasticity.

A notable finding was the ability of quercetin to increase BDNF levels in the PPAQ group. BDNF is essential for neuronal survival, growth, and plasticity, and its deficiency is associated with neurodegenerative diseases and cognitive deficits (Huang and Reichardt 2001; Scattoni et al. 2013). The increase in BDNF levels following quercetin treatment suggests that quercetin may support neuroplasticity and cognitive function, which are crucial for alleviating ASD symptoms (Arroyo-Brusé and Brusés 2012).

These findings are consistent with previous research highlighting the therapeutic potential of quercetin in various neurological disorders (D’Andrea 2015; Theoharides et al. 2012). Quercetin exhibits a multifaceted approach to neuroprotection by reducing oxidative stress and inflammation while enhancing neurotrophic support (Brigida et al. 2017). This study adds to the growing body of evidence supporting the use of dietary flavonoids such as quercetin in the management of neurodevelopmental disorders such as ASD (Theoharides et al. 2016; Taliou et al. 2013). Our study demonstrates that quercetin significantly reduces oxidative stress and inflammation, preserves neuronal integrity, and improves social and cognitive behaviors in a PPA-induced autism model in rats. These findings suggest that quercetin may hold promise as a therapeutic agent for ASD. Future studies should investigate the long-term effects of quercetin and its potential clinical applications in the treatment of ASD.

## Conclusion

This study investigated the neuroprotective effects of quercetin in a PPA-induced autism model in rats. The results showed that quercetin significantly reduced oxidative stress, as evidenced by lower MDA levels, and reduced inflammation by reducing proinflammatory cytokines such as TNF-α and IL-13. These findings suggest that the antioxidant and anti-inflammatory properties of quercetin contribute to its neuroprotective effects, as demonstrated by the preservation of neuronal integrity and increased BDNF levels.

Behavioral assessments revealed that quercetin-treated rats exhibited improved social interaction, exploratory behavior, and cognitive function. These improvements suggest that quercetin may help alleviate some of the core symptoms of ASD, including social deficits and cognitive impairments.

Overall, the study provides strong evidence that quercetin has potential as a therapeutic agent for ASD by targeting key pathological mechanisms such as oxidative stress, inflammation, and neuronal health. Future research should focus on the evaluation of the long-term effects of quercetin and its clinical applications to further confirm its potential in the treatment of ASD.

## Data Availability

No datasets were generated or analysed during the current study.
