# Correction: Antioxidant‑Effective Quercetin Through Modulation of Brain Interleukin‑13 Mitigates Autistic‑Like Behaviors in the Propionic Acid‑Induced Autism Model in Rats

**DOI:** 10.1007/s11481-026-10310-0

**Published:** 2026-08-20

**Authors:** Kubilay Doğan Kılıç, Gökçen Garipoğlu, Burak Çakar, Yiğit Uyanıkgil, Oytun Erbaş

**Affiliations:** 1 Faculty of Medicine, Department of Histology and Embryology, Ege University, İzmir, Türkiye; 2 Institute for Tissue Engineering and Regenerative Medicine, Helmholtz Zentrum München, Munich, Germany; 3 Museum Für Naturkunde, Leibniz Institute for Evolution and Biodiversity Science, Berlin, Germany; 4 Faculty of Health Sciences, Department of Nutrition and Dietetic, Bahçeşehir University, Istanbul, Türkiye; 5 Faculty of Medicine, Department of Histology and Embryology, İstinye University, İstanbul, Türkiye; 6 Cord Blood Cell – Tissue Research and Application Center, Ege University, İzmir, Türkiye; 7 Faculty of Medicine, Biruni Research Center (BAMER), Biruni University, Istanbul, Türkiye

**Correction to: Journal of Neuroimmune Pharmacology**

10.1007/s11481-025-10190-w

Following publication of the original article, the author identified an error in the caption of Fig. 4. The figure panels and the associated neuronal-count data are correct; however, the figure caption incorrectly referred to the cerebellum and Purkinje cell layers instead of the hippocampal CA1 and CA3 regions. This error does not affect the results, statistical analyses, interpretation, or conclusions of the article.

The correct Fig. 4 caption should appear as follows:

**Fig. 4** H&E Staining and Neuronal Counts in the Hippocampal CA1 and CA3 Regions. Control group male rats show preserved hippocampal architecture, intact pyramidal cell layers, and a high neuronal density. (A1) At 10× magnification, the control group shows the general architecture of the hippocampus, while (A2) at 40× magnification in CA1 and (A3) at 40× magnification in CA3, neurons exhibit intact nuclei and preserved cellular morphology. The PPA+Saline (PPAS) group male rats exhibit reduced neuronal density and morphological alterations associated with PPA-induced neuronal damage (B1). At 40× magnification, the PPAS group shows reduced neuronal density and disrupted cellular organization in CA1 (B2) and CA3 (B3). The PPA+Quercetin (PPAQ) group male rats show preservation of hippocampal architecture and neuronal density compared with the PPAS group (C1). At 40× magnification, quercetin treatment preserves neuronal morphology and cellular organization in CA1 (C2) and CA3 (C3), indicating its neuroprotective effects against PPA-induced hippocampal damage. Quantitative neuronal counts are presented in Table 2. Scale bars represent 200 μm in panels A1, B1, and C1 and 20 μm in panels A2–A3, B2–B3, and C2–C3.

The original article has been corrected.

